# Screen for ISG15-crossreactive Deubiquitinases

**DOI:** 10.1371/journal.pone.0000679

**Published:** 2007-07-25

**Authors:** André Catic, Edda Fiebiger, Gregory A. Korbel, Daniël Blom, Paul J. Galardy, Hidde L. Ploegh

**Affiliations:** 1 Program in Immunology, Harvard Medical School, Boston, Massachusetts, United States of America; 2 Whitehead Institute for Biomedical Research, Massachusetts Institute of Technology, Cambridge, Massachusetts, United States of America; 3 GI Cell Biology, Children's Hospital, Boston, Massachusetts, United States of America; 4 Merck, Rahway, New Jersey, United States of America; 5 Mayo Clinic, Pediatric and Adolescent Medicine, Rochester, Minnesota, United States of America; University of Munich, Germany

## Abstract

**Background:**

The family of ubiquitin-like molecules (UbLs) comprises several members, each of which has sequence, structural, or functional similarity to ubiquitin. ISG15 is a homolog of ubiquitin in vertebrates and is strongly upregulated following induction by type I interferon. ISG15 can be covalently attached to proteins, analogous to ubiquitination and with actual support of ubiquitin conjugating factors. Specific proteases are able to reverse modification with ubiquitin or UbLs by hydrolyzing the covalent bond between their C-termini and substrate proteins. The tail regions of ubiquitin and ISG15 are identical and we therefore hypothesized that promiscuous deubiquitinating proteases (DUBs) might exist, capable of recognizing both ubiquitin and ISG15.

**Results:**

We have cloned and expressed 22 human DUBs, representing the major clades of the USP protease family. Utilizing suicide inhibitors based on ubiquitin and ISG15, we have identified USP2, USP5 (IsoT1), USP13 (IsoT3), and USP14 as ISG15-reactive proteases, in addition to the *bona fide* ISG15-specific protease USP18 (UBP43). USP14 is a proteasome-associated DUB, and its ISG15 isopeptidase activity increases when complexed with the proteasome.

**Conclusions:**

By evolutionary standards, ISG15 is a newcomer among the UbLs and it apparently not only utilizes the conjugating but also the deconjugating machinery of its more established relative ubiquitin. Functional overlap between these two posttranslational modifiers might therefore be more extensive than previously appreciated and explain the rather innocuous phenotype of ISG15 null mice.

## Introduction

Posttranslational modification by ubiquitin regulates processes such as proteasomal degradation, intracellular trafficking, and transcription. Ubiquitin is attached to substrates in covalent isopeptide linkage or as an N-terminal fusion [1]–[3]. Ubiquitination, however, is reversible: the ubiquitin moiety can be released from substrates through the action of deubiquitinating proteases, which may rescue ubiquitinated substrates from their degradative fate [4]. In contrast, proteasome-associated DUBs enhance the rate of proteasomal degradation by removing bulky poly-ubiquitin chains from substrate proteins prior to proteolysis. Such DUBs enhance the processivity of the proteasome toward target proteins, and also recycle ubiquitin, a modifier that itself turns over slowly [5], [6]. DUBs are furthermore required to hydrolyze the ubiquitin precursor and generate the active ubiquitin monomer. Inspection of mammalian genomes shows the presence of more than 100 genes that encode putative DUBs, consistent with their specific and diverse regulatory functions. Ubiquitin-specific proteases (USPs) are the dominant family among DUBs [7].

Ubiquitin-like molecules show sequence and structural similarity to ubiquitin. Unlike ubiquitination, modification by UbLs usually does not target proteins for destruction by the proteasome. A notable exception may be FAT10, a modifier that serves as a ubiquitin-independent signal for proteasomal degradation [8]. The conjugation of UbLs to target proteins follows reaction pathways similar to those involved in ubiquitination [9]. The enzymes that attach or cleave UbLs are generally distinct from the ligases or proteases of the ubiquitin pathway.

A closely related homolog of ubiquitin in vertebrates is the UbL polypeptide ISG15, an interferon-inducible gene product that is strongly upregulated following viral or bacterial infection [10]. However, the molecular and regulatory consequences of ISGylation remain unknown [11]. ISG15 consists of two ubiquitin domains in a tandem arrangement, similar to FAT10. Unlike other members of the UbL family, ISG15 co-opts at least one of ubiquitin's conjugating enzymes, Ubc8 [12], [13] and the ubiquitin ligase Herc5 [14]–[17]. USP18 constitutes the only presently appreciated isopeptidase specific for ISG15, and its absence has profound effects on innate immunity, leading to increased resistance to certain viral infections [18], [19]. Notably, these effects appear not to be contingent upon proteolytic activity of USP18 [20], [21]. Apart from USP18, additional proteases for ISG15 must exist, since the ISG15 precursor protein is cleaved properly in USP18 knockout mice [19].

The C-terminal six amino acids of ubiquitin and ISG15 are identical. This tail region is required for specific recognition of ubiquitin by conjugating enzymes, and also for recognition of ubiquitin adducts by isopeptidases [22], [23]. The overlap in conjugation between ubiquitin and ISG15, as well as their C-terminal similarity, imply the existence of promiscuous DUBs, capable of removing both ubiquitin and ISG15 from substrate proteins. Here, we report on the identification of new ISG15-specific proteases measured by reactivity toward active-site directed probes and isopeptide-linked substrates [24]–[26].

## Results

### Activity-based profiling of DUBs

**Figure 1:**
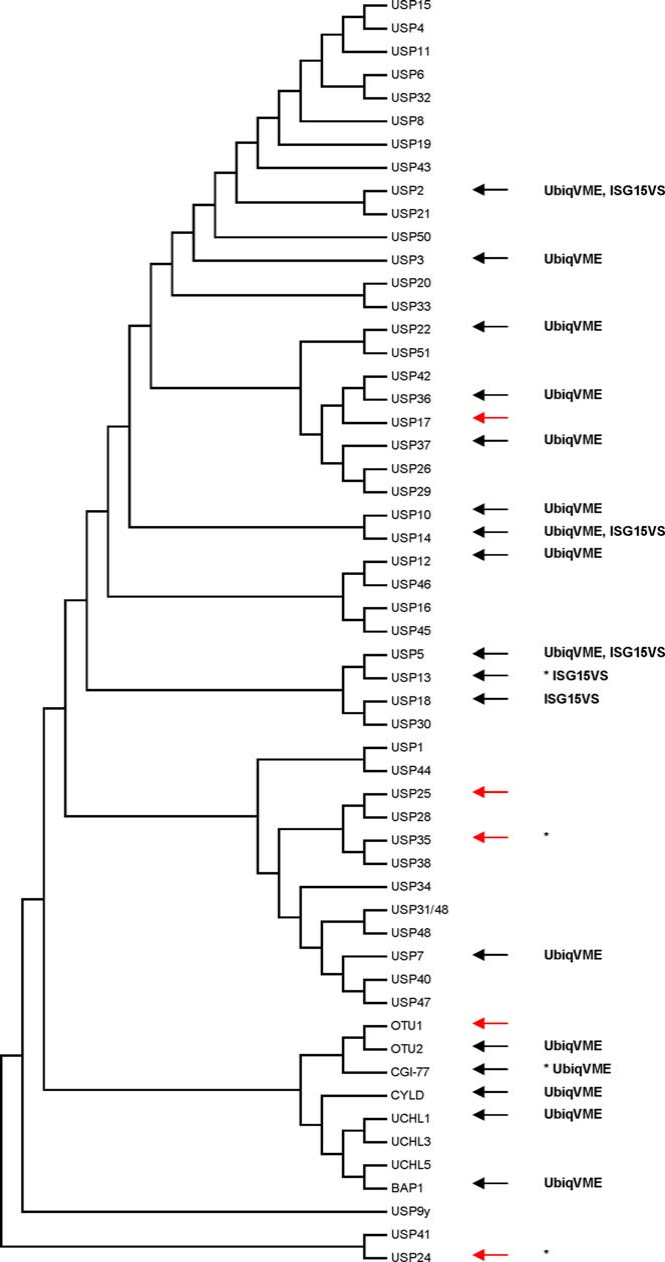
Phylogram representation of DUBs. Indicated with arrows are DUB homologs that were cloned and expressed by *in vitro* transcription/translation (IVT). Red arrows depict DUBs that bound to neither probe (UbVME, ISG15VS, or SUMO1VS), whereas black arrows indicate DUBs that formed covalent adducts with the indicated probes. Our screen represents the first biochemical proof for protease activity of USP13 and the Otubain-homolog CGI-77 (DUB homologs without publication record regarding biochemical function are marked with an asterisk). Otubain1 (OTU1) is an exception in that it binds to alkylhalide- or aldehyde-based probes, but not to the Michael acceptors employed in this study (data not shown).

**Figure 2:**
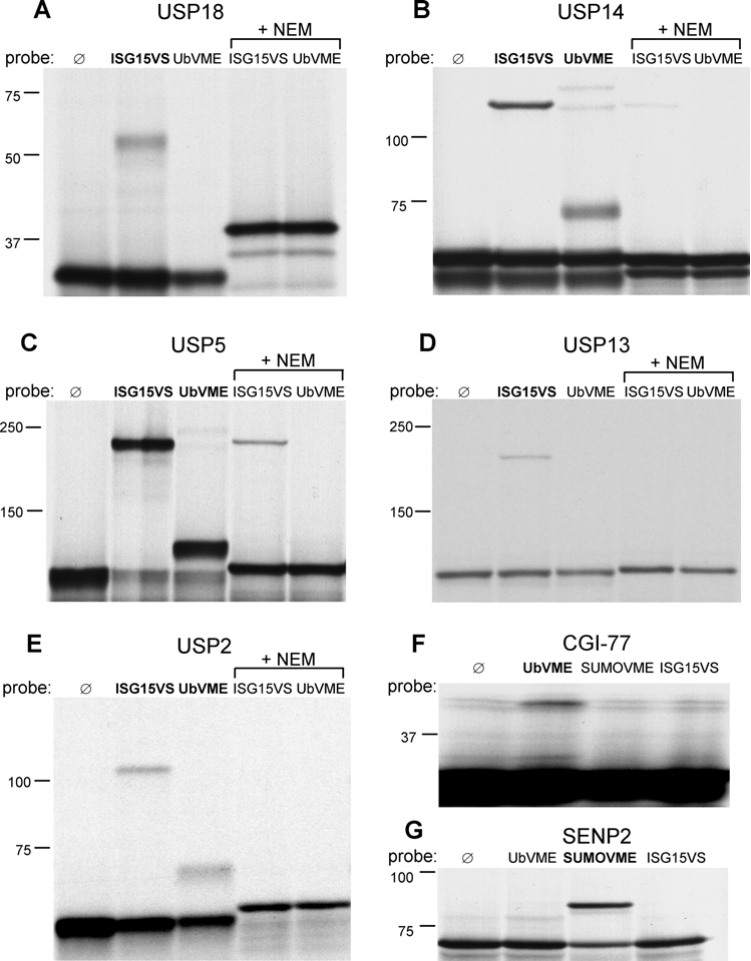
Activity-based profiling of DUBs. IVT was used to generate DUBs for profiling activity toward ISG15VS, UbVME and SUMO1VME probes. After incubation with the probes, samples were analyzed by SDS-PAGE, as shown for USP18 (A), USP14 (B), USP5 (C), USP13 (D), USP2 (E), and CGI-77 (F). Binding was inhibited by preincubation of the proteases with NEM. The SUMO protease SENP2 interacts specifically with SUMO1VME (G). Molecular weight in kDa is indicated on the left.

Figure 1 shows a consensus phylogram based on the alignment of catalytic core sequences of DUBs, including the majority of known human USP homologs. In this tree, the ISG15-protease USP18 clusters close to USP5 (IsoT1) and its isoform USP13 (IsoT3). Previous work had identified USP5 as a protease with affinity for both ubiquitin [27] and ISG15, as shown by its reaction with an electrophilic ISG15 derivative, ISG15-vinyl sulfone (ISG15VS) [28]. To probe for additional ISG15-reactive proteases, we have cloned and expressed a total of 22 human DUB homologs from different clades of this phylogram (indicated with arrows), 17 of which reacted with a ubiquitin-based probe and/or an ISG15-based probe (see below). The screen was based on *in vitro* transcription and translation (IVT) of cloned cDNAs, which affords a rapid method to generate radiochemically pure proteins. This technique allows the generation of DUBs that cannot be readily expressed in bacterial systems, or that are sequestered in subcellular compartments or in multimolecular complexes when expressed in cell lines. To profile for DUB specificity, we used recombinantly expressed ubiquitin, SUMO1 and ISG15, and installed an electrophilic trap at their C-terminus to obtain the active-site probes ubiquitin-vinylmethyl ester (UbVME), SUMO1VME and ISG15VS, respectively [28]. DUBs generated by IVT were incubated with each of these three probes (Figure 2), followed by direct analysis of the reaction mixture by SDS-PAGE. We have determined by X-ray crystallography that probes of this type form a covalent adduct with the catalytic cysteine residue of active DUBs to yield a thioether-linked adduct between enzyme and probe [26]. When unmodified IVT products are run adjacent to samples incubated with these activity-based probes, the adduct is readily detected through a shift in apparent molecular mass. USP2, USP5, USP13 and USP14 reacted with ISG15VS (Figure 2B, C, D, E).

The following observations confirm the validity of our assay. First, the *bona fide* ISG15-isopeptidase USP18 displayed reactivity only towards ISG15VS (Figure 2A), whereas most of the DUBs reacted only with UbVME. An example is shown with CGI-77 (Figure 1, 2F), a previously uncharacterized Otubain-homolog. Second, as a negative control, the SUMO protease SENP2 formed an adduct exclusively with SUMO1VME (Figure 2G). We found no evidence for any of the DUBs evaluated here to display reactivity toward the SUMO1 probe (not shown). The presence of the reactive group alone is clearly not sufficient for binding to the active-site cysteine of a protease and specificity of a DUB thus depends on the peptide moiety of the probe, containing either ubiquitin, SUMO1 or ISG15. Lastly, all covalent modifications of DUBs by active-site directed probes were blocked by pretreatment of the translated polypeptides with the sulfhydryl alkylating agent N-ethylmaleimide (NEM) (Figure 2), confirming the cysteine-dependency of adduct formation.

### Characterization of the abnormal size shift in SDS-PAGE

**Figure 3:**
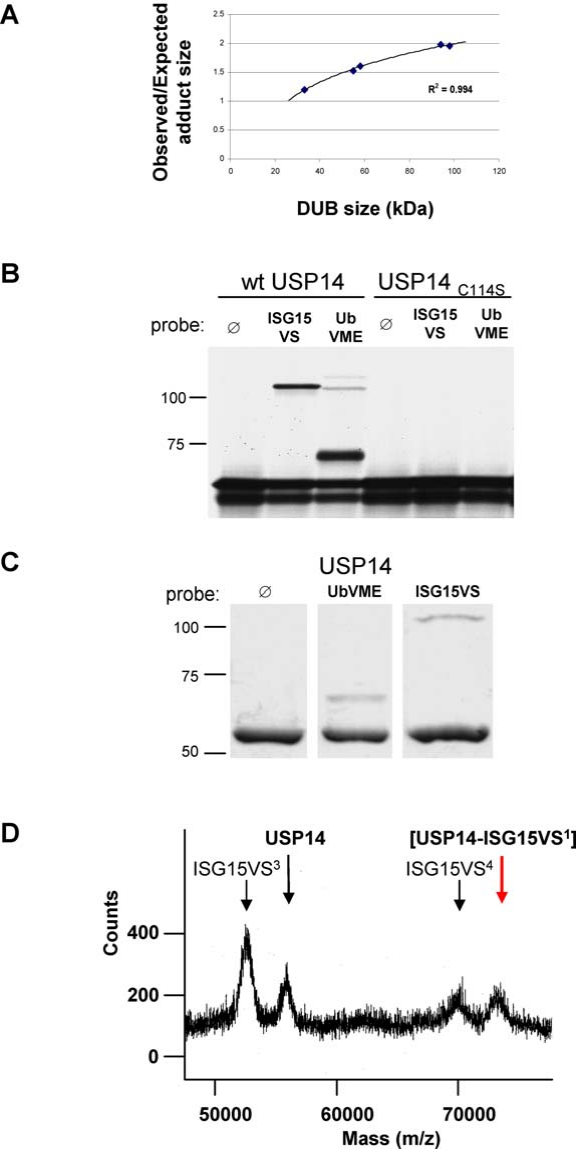
Unexpected apparent molecular mass of ISG15VS-DUB adducts is caused by unusual behavior in SDS-PAGE. (A) Plot showing the ratio of observed versus expected ISG15VS-DUB adduct size of USP2, USP5, USP13, USP14 and USP18 in SDS-PAGE (8%) over the size of the unmodified DUBs. (B) Mutation of the catalytic cysteine residue to serine (C114S) in USP14 abolishes its reactivity toward UbVME and ISG15VS. When stored for longer periods at room temperature, the probes polymerize covalently, presumably by formation of secondary amine bonds between internal lysine residues and the reactive Michael acceptor at the C-terminus, thus resembling isopeptide-linked polyubiquitin. Such polymeric probes of UbVME likely caused the additional high-molecular mass adducts observed for USP14. Note that the smallest version of these adducts (a UbVME dimer) has a maximum electrophoretic mobility similar to that of the diubiquitin-like ISG15VS when complexed to USP14. The absence of any adducts in the C114S mutant of USP14 excludes the possibility of multiple binding sites for the probes. (C) Purified recombinant USP14 labels with UbVME and ISG15VS and results in the same abnormal mobility shift for ISG15VS-USP14 as seen in the IVT labeling experiments. (D) MALDI-TOF mass spectroscopic analysis of USP14 after incubation with ISG15VS. As described above for UbVME, ISG15VS also engages in internal polymerization. Molecular masses consistent with tri- and tetrameric ISG15VS are marked in this spectrogram by the numbers in superscript. Monovalently modified USP14 results in an adduct of predicted size, indicated with a red arrow. This complex is unique to the mixture containing both USP14 and ISG15VS, and is absent in the mass spectra of either component alone (data not shown).

In accordance with previous results [28], we observed a non-linear decrease in electrophoretic mobility of the ISG15VS adducts. The ISG15 probe has a mass of 17.4 kDa, whereas the size increase observed for each of the DUBs investigated here is in the order of 25–110 kDa, when bound to ISG15VS. The correlation between the abnormal shift and the initial mass of the unmodified DUB suggests that the decrease in gel mobility is based on steric properties of the branched adduct, and is not caused by covalent modification of a single DUB by multiple ISG15VS molecules. In fact, in our sample set, the observed size increase of the ISG15VS-DUB adducts based on SDS-PAGE very closely matched a logarithmic equation (Figure 3A and see Methods). To further verify that DUBs are modified by only a single ISG15VS probe per molecule, we replaced the catalytic cysteine at position 114 in USP14 with a serine residue. As expected, this mutation abolished all labeling (Figure 3B). Our assay was conducted in IVT lysate and the size increase of the ISG15VS adduct could potentially reflect modification of USP14 by additional factors. However, even USP14 that was recombinantly expressed in bacteria and >95% pure showed the same abnormal electrophoretic mobility for its ISG15VS adduct (Figure 3C). Mass spectrometry confirmed the monovalent modification of purified USP14 by ISG15VS and excluded covalent binding of additional factors to the complex (Figure 3D). Collectively, these experiments establish that the observed shift in apparent molecular mass of the ISG15VS-DUB adducts is solely a consequence of its unusual electrophoretic behavior. It also underscores the uncertainties in estimating the degree of modification of a target protein with UbLs by SDS-PAGE alone.

**Figure 4:**
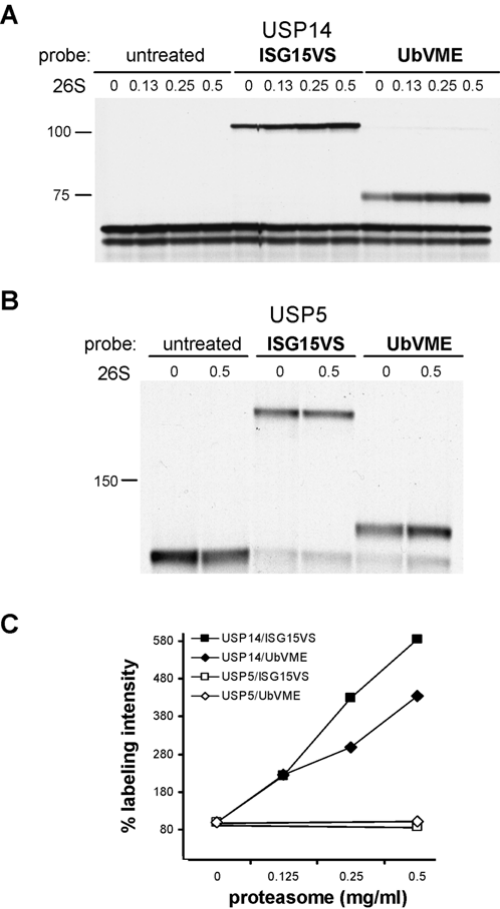
Reactivity of USP14 toward ISG15VS is augmented by proteasomal association. USP14 and USP5 were generated by IVT. Their activity toward ISG15VS and UbVME was analyzed in the presence of increasing concentrations of purified human 26S proteasomes. (A) Activity of USP14 toward UbVME and ISG15VS increases as a function of the concentration of added purified 26S proteasomes (in mg/ml). (B) The activity of USP5 remains unaffected. (C) Quantification of the radioactive signal of covalently modified USP5 and USP14. Binding affinity is depicted on the y-axis as percent in labeling intensity, determined by the ratio of labeled versus unlabeled USP5 or USP14. The ratio in the absence of exogenous proteasomes is defined as 100%.

### USP14 reacts more efficiently with ISG15VS in its proteasome-associated form

USP14 and its yeast counterpart Ubp6 show significantly higher activity when bound to the 26S proteasome [29]. This activity may in fact be strictly dependent on association with the proteasome, as shown for Ubp6 [30]. As further evidence for a physiological role of the interaction of USP14 with ISG15, we investigated whether the allosteric activation of USP14 also influences its reactivity toward ISG15VS. We examined labeling of USP14 with the ubiquitin- and the ISG15-based probes as a function of the concentration of added purified proteasomes. As a negative control, we evaluated USP5, a DUB that is not a known interaction partner of the 26S complex *in vivo*. As anticipated, the inclusion of purified proteasomes had no effect on the ISG15VS- or UbVME-reactivity of USP5 (Figure 4B). In contrast, we observed a dose-dependent increase in ISG15VS adduct formation of USP14 with increasing proteasome concentration, indicating enhanced activity of this DUB. The effect was similar in magnitude to that seen for the ubiquitin probe (Figure 4A, C).

### USP14 is an isopeptidase only in association with the proteasome

**Figure 5:**
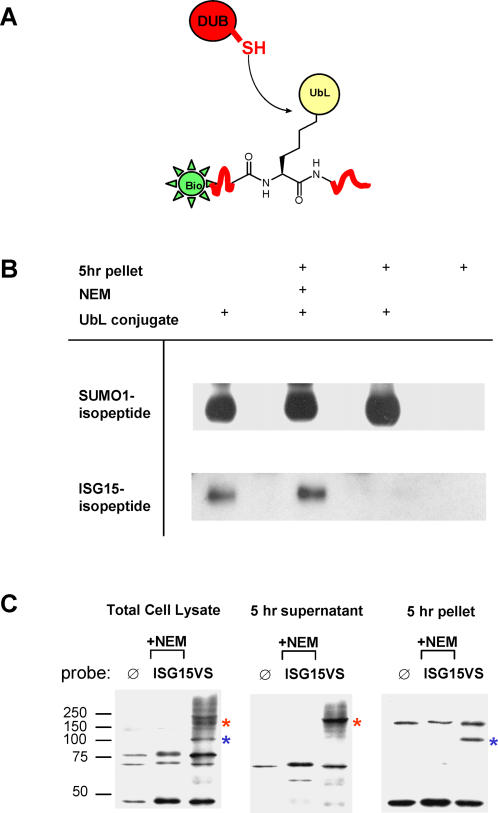
Proteasome-associated USP14 has ISG15-specific isopeptidase activity. (A) Scheme depicting the UbL-peptide conjugate used to assay isopeptidase activity. The biotinylated peptide heptamer is attached to either ISG15 or SUMO1 in isopeptide-linkage. Upon hydrolysis of the isopeptide bond by a specific DUB, the heptamer is released and the biotin signal lost. (B) Incubation of proteasome-enriched fraction (“5 hr pellet”) with UbL-peptide conjugate. After overnight incubation, the ISG15-peptide conjugate is completely cleaved, resulting in loss of the biotin-signal (significant proteolysis occurs already after one hour, data not shown). This activity is sensitive to NEM. Hydrolysis is not observed for the SUMO1-peptide conjugate. (C) Anti-HA immunoblot of HA-ISG15VS treated subcellular fractions. Based on previous identification [28] and on electrophoretic mobility, USP5 is the dominant ISG15-reactive DUB in the five-hour supernatant, which is enriched for uncomplexed proteins of light and moderate size (red asterisk). The five-hour pellet represents heavy cytosolic complexes, in particular the 26S proteasome, and contains USP14 as the only ISG15-reactive DUB (blue asterisk).

While recombinant USP14 in its purified form bound to electrophilic probes (Figure 3C), we did not detect robust hydrolytic activity against ubiquitin-AMC or against ubiquitin- or ISG15-linked isopeptide fusion proteins (data not shown). However, using sequential ultracentrifugation to obtain a cytosolic fraction that is enriched in 26S proteasomes [29], we could show that proteases in this fraction efficiently and specifically cleave an ISG15-isopeptide linked substrate (Figure 5A, B). The absence of proteolytic intermediates suggests specific cleavage of the isopeptide bond. In addition, the same bait peptide linked to SUMO1 was stable and not hydrolyzed, even upon prolonged incubation for over 24 hours with the proteasome fraction. Proteolysis of the ISG15-linked peptide substrate was inhibited by inclusion of NEM, indicating cleavage by cysteine proteases. Analysis by reaction with ISG15VS supports that USP14 is the only active ISG15-specific protease in the proteasome-enriched fraction (Figure 5C). While we cannot formally exclude the possibility of a yet undefined enzyme binding to ISG15VS, we consider this unlikely: such a protease would have to display a mass highly similar to that of USP14 and, furthermore, it would have to sediment after centrifugation for 5 hours at 100,000 g. However, only few deubiquitinating enzymes are sedimentable, none at a level comparable to USP14 [29], [31].

### Activity in cell lysate and subcellular distribution of DUBs

**Figure 6:**
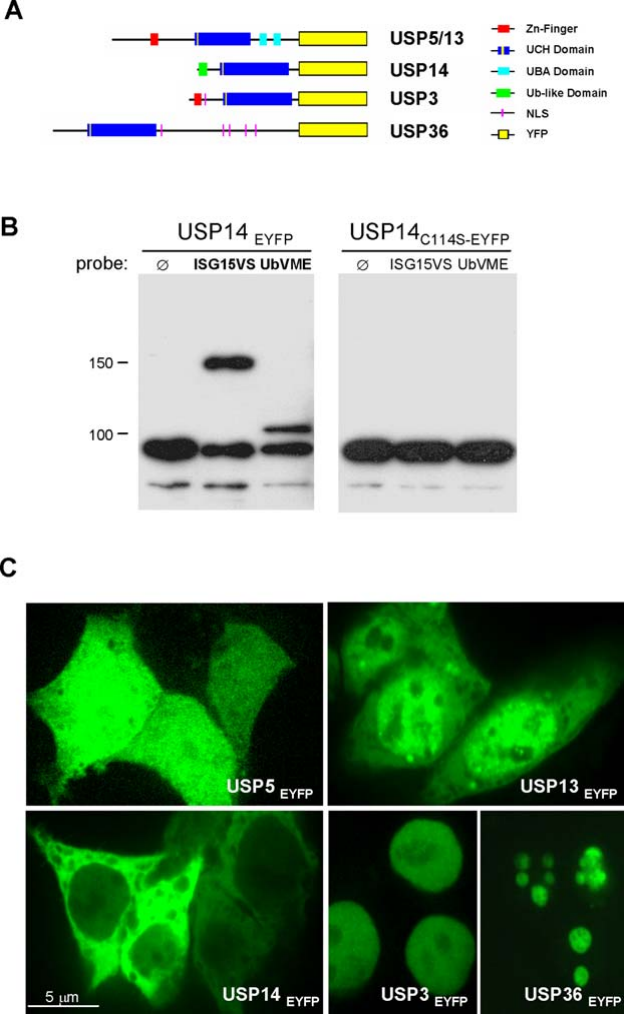
*In vivo* probe-binding studies and analysis of subcellular DUB localization. (A) Schemes of the EYFP fusion proteins: UCH-like Zinc Finger Motif (red), proteolytic UCH domain (blue, with the putative active-site cysteine depicted in yellow), ubiquitin-binding UBA-domain (light blue), ubiquitin-like domain (green), putative nuclear localization sequence (pink), EYFP fusion protein (yellow box). (B) *In vivo* binding of ubiquitin and ISG15 probes. Lysates obtained from 293T cells transfected with USP14_EYFP_ or USP14_C114S-EYFP_ were reacted with UbVME or ISG15VS and compared to untreated aliquots in an anti-YFP immunoblot. USP14_EYFP_ but not USP14_C114S-EYFP_ reacts with the probes. (C) Subcellular localization of DUBs analyzed via the distribution of C-terminal EYFP fusions. 24 hours post-transfection, 293T cells were fixed with paraformaldehyde and analyzed by confocal fluorescence microscopy. USP5_EYFP_ can be found throughout the cell (upper left). USP13_EYFP_ is expressed mainly within the nucleus, in a speckled pattern (upper right panel). USP14_EYFP_ is detected predominantly in the cytoplasm, with lower levels in the nucleus (lower left panel). USP3_EYFP_ and USP36_EYFP_ (lower right panel) are detected in the nucleus and nucleolus, respectively.

The wealth of USPs found in the human proteome likely reflects substrate specificity, but potentially also complementation in terms of expression profiles and subcellular distribution. We therefore sought to analyze the intracellular distribution pattern of a subset of our crossreactive DUBs, using confocal microscopy. The analysis of a genome-wide set of C-terminal GFP fusion proteins for yeast had shown remarkably few with altered function or subcellular distribution (<5%), validating the choice of such C-terminal modifications [32]. Using anti-G/YFP antibodies, we also utilized this tag to assay for activity of DUBs in cell lysate. We cloned and transiently expressed five USP-EYFP constructs in 293T cells: USP5, USP13, USP14, USP3, and USP36 (Figure 6A). Lysate of USP14_EYFP_ transfected cells was incubated with the ubiquitin and the ISG15 probe, and assayed by anti-YFP immunoblot analysis (Figure 6B). Whereas the USP14_EYFP_ construct reacted with both probes, the respective C114S mutant did not, in agreement with the results of our IVT screen. With respect to subcellular distribution, we were particularly interested in the expression pattern of USP5 and USP13, given that these two isoforms displayed different specificity in UbVME and ISG15VS labeling experiments. USP5_EYFP_ was found throughout the cell (Figure 6C, upper left panel), similar to USP18 [33]. In contrast, its close relative USP13_EYFP_ was expressed mainly in the nucleus in a speckled pattern (Figure 6C, upper right panel). Consistent with the *in vivo* interaction between USP14 and the proteasome [5], we observed USP14_EYFP_ predominantly in the cytoplasm, though we also noticed fluorescence in the nucleus (Figure 6C, lower left panel). To demonstrate that the presence of the C-terminal YFP fusion does not interfere with the endogenous distribution of these enzymes, we analyzed USP3_EYFP_ (Figure 6C, lower right panel) and USP36_EYFP_ (Figure 6C, lower right panel). USP3 is predicted to be a nuclear protein [34] and USP36 was identified as a nucleolar protein [35]. Both proteins were detected in the expected subcellular compartment. Combined, these results indicate that ISG15-specific proteases are expressed throughout the cell – a feature also proposed for DUBs. This observation supports the notion that unlike SUMOylation, which is believed to mostly occur in the nucleus [36], ISG15-modification affects many cellular compartments.

## Discussion

**Figure 7:**
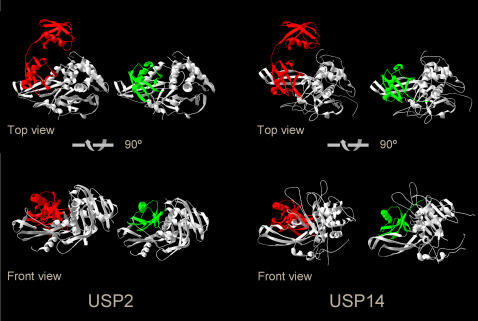
Structures of USP2 and USP14, modeled with ubiquitin and ISG15. Both USP2 and USP14 accommodate ubiquitin (green) in a shallow groove, measuring approximately 25–30 Å in diameter (view from top shown in the upper panels, view from front shown in the lower panels). Based on these complexes, the C-terminal domain of ISG15 (red) can be modeled into the groove to replace ubiquitin. The N-terminal domain of ISG15 is not involved in the hydrolysis reaction, and is located outside of the catalytic core domain of both proteases. The N-termini of USP2 and USP14 are lacking in these representations and extend to the right side of the structure models.

Our data show the existence of multiple ISG15-reactive DUBs, a finding that further strengthens the similarities between ubiquitin and ISG15. USP2 is a highly active protease [37] and it represents one of only few mammalian DUBs with a known target. USP2 exhibits oncogenic potential in prostate cancer by stabilizing its substrate Fatty Acid Synthase (FAS) [38], and FAS has indeed been identified as a target of ISG15 modification [39]. Furthermore, USP2 has been implicated in the regulation of the p53 pathway [40]. The recently solved structures of USP2 and USP14 [41], [42] show that both proteases accommodate the ubiquitin molecule in a shallow pan-like protrusion. Based on the orientation of the ubiquitin protein in both structures, ISG15 easily fits into the catalytic domain of USP2, USP14, and USP5 (data not shown) without apparent steric clashes (Figure 7) [23], [43].

Stimulation by interferons alters the composition of the proteolytic proteasome core [44], tailoring its activity toward generation of peptide-MHC complexes for inspection by the immune system. Interferon treatment also results in enhanced modification of proteins by ISG15 - a factor that evolved in the vertebrate lineage, and whose origin thus coincides with that of the adaptive immune system. Interestingly, inhibition of the proteasome leads to rampant accumulation of ISG15-modified substrates [45]. While nothing is known about the molecular functions of ISG15, its structural relative FAT10 is a modifier that destines proteins for degradation by the proteasome [8]. We now have demonstrated that USP14 exhibits proteasome-associated isopeptidase activity toward ISG15. Could therefore ISG15 be a (co-)modifier of proteins destined for proteasomal degradation? We have found no evidence that USP14 markedly changes the amount of ISG15-modified substrates in cells (data not shown). However, USP14 is not a vital protease [30], [46] and its low catalytic turnover does not affect overall ubiquitin conjugation either [47]. A recent study suggests that USP14 might have a more complex role, by inhibiting the proteasome in addition to acting as a deubiquitinase [48].

The close sequence relationship between USP5 and USP13 (61.4% identity, 26.9% similarity) is contrasted by the functional differences and localization of these two proteases. These enzymes provide a unique opportunity to investigate the structural features that may contribute to ubiquitin versus ubiquitin-like specificity. A characteristic of USP5 and USP13 is the tandem occurrence of a UBA domain, which has been implicated in the binding of ubiquitin [49]. Our results raise the possibility that UBA domains in general interact not only with ubiquitin, but also with ISG15. Alternatively, ISG15 with its multiple lysines could act as a ubiquitination anchor, and USP5 may be a protease responsible for depolymerization of such chains [50], [51]. Moreover, the C-terminal hydrolase that processes the ISG15 precursor has not been identified yet, and any of the novel ISG15-specific proteases described here are potential candidates.

### Conclusion

The ubiquitin gene is prone to duplications and insertions, leading to the formation of new fusion proteins [52], [53]. ISG15 likely emerged approximately 400–600 million years ago, when a nucleotide stretch from the polyubiquitin precursor gene, encoding a ubiquitin-dimer, was accidentally inserted in an area of the genome that was or that came under control of an interferon promoter. From an agnostic point of view, one could argue that ISG15 simply has no relevant function. The moderate or absent phenotype of the ISG15 knockout in mice [11], the fact that ISG15 has not (yet) established its very own family of conjugating and deconjugating enzymes, and ISG15's relatively low degree of conservation between species would all support this view. Yet, ISG15's massive expression upon interferon challenge [54] likely reflects a role in anti-microbial or anti-viral defense [55]–[59]. And adaptation to the specific needs of host immunity often demands polymorphism. As a result, some genes most critical to the immune response are paradoxically least conserved. For example, cytokines and cytokine receptors substantially differ between species [60] and it is interesting to note that ISG15 and ubiquitin were initially reported to be cytokines [61], [62]. Similar observations were made for the ubiquitin-like modifier FUBI (also known as Fau or MNSFβ) [63], [64]. If true, how do these factors gain access to the secretory pathway? It may pay to approach ISG15 from a less conventional perspective and from this vantage point, we might uncover new functions of ubiquitin as well.

## Methods

### DNA constructs

USP2 and USP18 cDNAs were cloned from a human kidney cDNA library (BioChain Institute, Inc.). The cDNAs encoding the other human DUBs were obtained from ATCC. All cDNAs encoding full-length DUBs were subcloned into pcDNA3.1. Point mutations were generated with the Stratagene “Quik Change II” kit. The Genbank Identification numbers of the cloned constructs are: USP2 (GI:21361712), USP3 (GI:10720340), USP5 (GI:4507855), USP7 (GI:4507857), USP10 (GI:24307889), USP12 (GI:32698815), USP13 (GI:4507849), USP14 (GI:4827050), USP17/DUB3 (GI:37778800), USP18 (GI:32313610), USP22 (GI:113426698), USP24 (GI:113408685), USP25 (GI:6941888), USP35 (GI:89034166), USP36 (GI:35250686), USP37 (GI:32698744), Bap1 (GI:4757836), CylD (GI:14165258), CGI-77 (GI:40254879), OTU1 (GI:109148508), OTU2 (GI:12962939), UCH-L1 (GI:93279272).

### Bioinformatics

The protein sequences of human DUBs were obtained from the National Library of Medicine and the core domains were aligned with the CLUSTALW algorithm (EBI server) [65] and manually edited with Genedoc (http://www.psc.edu/biomed/genedoc/) by K.B. Nicholas & HB Nicholas Jr., using the putative active-site cysteine as an alignment anchor. The phylogram represents a consensus tree based on 100 bootstrap iterations, calculated by the Minimum Evolution method under default parameters [66].

### *In vitro* transcription and translation (IVT)

IVT was performed using the “TNT-T7 Quick Reticulocyte Lysate System” kit (Promega) for 30 to 45 min (0.25–1 µg DNA per reaction). Then, aliquots of the reaction mix were treated with RNase B (1 mg/ml, Sigma) for 10 min and incubated with the probes as described below. SDS-PAGE followed by fluorography was performed as described [67].

### Labeling with ubiquitin and UbL electrophilic probes

The synthesis of human ubiquitin and UbL probes has been described [24], [28]. IVT products were incubated with saturating amounts of the individual probes (0.2–0.4 µg/10 µl IVT lysate). Preincubation with NEM was performed for 10 min at room temperature at a final concentration of 10 mM, after RNase B treatment. Autoradiograms were subjected to quantification of the optical density with NIH Image software (version 1.32j) as ratio of labeled versus unlabeled IVT products. Purified human 26S proteasomes for the experiments in Figure 4 were purchased from Biomol International and inhibited with MG132 (50 µM). *E. coli*-expressed human recombinant USP14 for the experiments shown in Figure 3C and 3D was purchased from Boston Biochem. The observed size of ISG15VS-adducts in SDS-PAGE (8%) followed a logarithmic equation (R^2^>0.99):
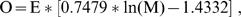
with O being the observed adduct size, E the expected adduct size, and M the mass of the unmodified DUB (for M>25 kDa).

### Synthesis of ISG15- and SUMO1-branched peptide conjugates

To 50 µL of conjugation buffer (100 mM Tris, pH 7.4, 5 mM MgCl_2_, 20 mM DTT, 40 mM ATP) was added ISG15 (Boston Biochem, 9.4 µM final) or GST-SUMO1 (Boston Biochem, 10.4 µM final) and biotinylated peptide 7-mer (biotin-VKAKIQD-OH, 250 µM final). The solution was mixed thoroughly and to this was added ISG15 activating enzyme (Boston Biochem, ISG15 E1, 50 nM final) and UbcH8 (Boston Biochem, 250 nM final), or SUMO activating enzyme (SAE1/SAE2 heterodimer, 50 nM final) and UbcH9 (250 nM final). The solution was mixed and incubated for 15 hours at 37°C. The reaction mixture was transferred to a microcentrifuge membrane filter (Vivascience, 5000 Da MWCO), diluted to 600 µL total volume with 50 mM Tris, pH 7.4 and concentrated at 4°C to 50 µL. This dilution/concentration procedure was repeated six times. The products were transferred to a clean tube and diluted with 50 mM Tris, pH 7.4 to a final volume of 100 µL. The SUMO1- and ISG15-linked biotinylated isopeptide was detected after transfer to a PVDF membrane (Perkin Elmer) with Streptavidin-HRP (Amersham).

### Subcellular fractionation and isopeptide cleavage assay

EL4 cells were lysed with glassbeads and the proteasome-enriched fraction was retrieved by consecutive ultracentrifugation steps as previously described [29]. Proteasome activity was inhibited with MG132 (50 µM). 10 µg of fraction protein were incubated with 0.2 µg of N-terminally HA-tagged ISG15VS in a total volume of 10 µl for two hours at room temperature to detect ISG15VS-reactive proteases by anti-HA immunoblotting. The cleavage assay for ISG15- or SUMO1-branched peptides was performed at 37°C using 20 µg of total protein from the proteasome-enriched fraction and 5 µl of branched peptides in a total volume of 10 µl. HA-ISG15VS treated subcellular fractions were analyzed with a monoclonal anti-HA antibody (12CA5). Immunoblotting was performed as published [67].

### Cell lines, transient transfections and anti-YFP immunoblotting

293T cells were maintained in DME medium as described [67]. Various constructs were expressed by transient transfection, using a liposome-mediated transfection protocol (5–10 µg of DNA/20 µl of Lipofectamine-2000 per 10 cm dish; Invitrogen) as described [67]. Cells were analyzed between 24 and 48 h after transfection. C-terminal EYFP fusion proteins of DUBs were generated by subcloning from pcDNA3.1 into pEGFP-N1 (Clontech). NP40 lysates of USP14_EYFP_ and USP14_C114S-EYFP_ transfected 293T cells were prepared and incubated with active-site probes as described [24]. Due to the high similarity with GFP, EYFP fusion proteins can be detected with a polyclonal anti-GFP rabbit serum [68]. Immunoblotting was performed as published [67].

### Confocal microscopy

Immunofluorescence experiments were performed as described [67] with minor modifications. Cells were allowed to attach to slides overnight. After fixation with 3.7% paraformaldehyde for 20 min at room temperature, subcellular localization of EYFP fusion proteins was analyzed with a Perkin Elmer spinning disk confocal microscope Ultraview RS system. The microscope used was a Nikon TE2000-U inverted unit with a Nikon 100× 1.4NA DIC lens. The imaging medium was Nikon type A immersion oil.

### Structure models

The structures of USP2 and USP14 in complex with ubiquitin and the structure of ISG15 were obtained from the PDB website (2HD5, 2AYO, 1Z2M) and ISG15 was manually modeled onto USP2 and USP14 with the SPDB-Viewer [69].
